# Age-dependent involvement of gut mast cells and histamine in post-stroke inflammation

**DOI:** 10.1186/s12974-020-01833-1

**Published:** 2020-05-19

**Authors:** Maria Pilar Blasco, Anjali Chauhan, Pedram Honarpisheh, Hilda Ahnstedt, John d’Aigle, Arunkumar Ganesan, Sriram Ayyaswamy, Frank Blixt, Susan Venable, Angela Major, David Durgan, Anthony Haag, Julia Kofler, Robert Bryan, Louise D. McCullough, Bhanu Priya Ganesh

**Affiliations:** 1 Department of Neurology, University of Texas McGovern Medical School, Houston, USA; 2 Department of Anesthesiology, Baylor College of Medicine, Houston, USA; 3 Department of Pathology and Immunology, Baylor College of Medicine, Houston, USA; 4 Department of Pathology, University of Pittsburg, Pittsburgh, USA

**Keywords:** Mast cells, Histamine, Histamine receptor, Cytokines, Post-stroke, Microbiome, Intestinal epithelium

## Abstract

**Background:**

Risk of stroke-related morbidity and mortality increases significantly with age. Aging is associated with chronic, low-grade inflammation, which is thought to contribute to the poorer outcomes after stroke seen in the elderly. Histamine (HA) is a major molecular mediator of inflammation, and mast cells residing in the gut are a primary source of histamine.

**Methods:**

Stroke was induced in male C57BL/6 J mice at 3 months (young) and 20 months (aged) of age. Role of histamine after stroke was examined using young (Yg) and aged (Ag) mice; mice underwent MCAO surgery and were euthanized at 6 h, 24 h, and 7 days post-ischemia; sham mice received the same surgery but no MCAO. In this work, we evaluated whether worsened outcomes after experimental stroke in aged mice were associated with age-related changes in mast cells, histamine levels, and histamine receptor expression in the gut, brain, and plasma.

**Results:**

We found increased numbers of mast cells in the gut and the brain with aging. Using the middle cerebral artery occlusion (MCAO) model of ischemic stroke, we demonstrate that stroke leads to increased numbers of gut mast cells and gut histamine receptor expression levels. These gut-centric changes are associated with elevated levels of HA and other pro-inflammatory cytokines including IL-6, G-CSF, TNF-α, and IFN-γ in the peripheral circulation. Our data also shows that post-stroke gut inflammation led to a significant reduction of mucin-producing goblet cells and a loss of gut barrier integrity. Lastly, gut inflammation after stroke is associated with changes in the composition of the gut microbiota as early as 24-h post-stroke.

**Conclusion:**

An important theme emerging from our results is that acute inflammatory events following ischemic insults in the brain persist longer in the aged mice when compared to younger animals. Taken together, our findings implicate mast cell activation and histamine signaling as a part of peripheral inflammatory response after ischemic stroke, which are profound in aged animals. Interfering with histamine signaling orally might provide translational value to improve stroke outcome.

## Introduction

Aging is a major risk factor for stroke, stroke-related mortality, and post-stroke complications [1]. Aging is associated with increased inflammation and changes in the immune response to injury [2, 3]. To date, few studies have investigated the effects of aging on peripheral immune responses after ischemic stroke. Histamine (HA) is a major mediator of acute inflammatory response to tissue injury [4–7]. Among HA-producing cells, mast cells (MCs) are a major source of HA in the early phases of inflammation [8–10]. MCs release stored and newly synthesized HA along with other inflammatory mediators such as proteases, cytokines, and chemokines [9, 11–16]. As the largest immune organ in the body, the gut is a major site of MC progenitors and HA production [17, 18]. Gut MC progenitors constitutively home to the intestinal mucosa [19, 20], and upon initiation of the inflammatory response, they are recruited out of the gut environment and mature in a HA-dependent manner [14]. The inflammatory cascade originating in the cerebral vasculature that leads to the systemic immune response by brain ischemia is a major factor in stroke pathophysiology and outcome [21–23].

Accumulating pre-clinical data suggests that stroke leads to a more significant disruption of gut homeostasis in aged mice when compared to young mice [24, 25], but the molecular mediators of these age-dependent changes are poorly defined. MCs are among the first-responders to tissue injury and secrete large amounts of HA to initiate a cascade of local and systemic inflammatory processes [10]. The diverse effects of HA are determined by the function, structure, tissue distribution, and ligand affinity of the four HA receptor subtypes (H1R through H4R) [8, 26]. In the gut, the majority of HA receptors are of H1R and H2R subtypes [27, 28].

Using global MC knockout models, one study reported that meningeal MCs worsen stroke-induced brain injury in murine models [29]. Given the limitation of global knockout models however, the role of gut MCs, HA, and HA receptors, where most progenitor MCs are found [18, 30], has not been evaluated after stroke. We hypothesized that in aged mice, stroke would induce enhanced peripheral inflammation and an increase in MCs and HA receptor activation in the gut. Therefore, we aim to examine peripheral gut mast cell activation and histamine signaling in response to stroke. Aged MCs are known to be in an increased state of activation [31]*.* In this work, we evaluated age-dependent changes in local and systemic HA levels, HA receptors, and gut MCs after stroke. Specifically, we investigated how the gut HA and HA receptor levels differ in response to stroke at 6 h, 24 h, and 7 days after experimental stroke and compared the response in young (Yg) and aged (Ag) mice. We found that MCs increase with age in both the gut and in the brain. Our data also showed that H2R expression, loss of gut barrier integrity, and elevated plasma HA and other pro-inflammatory cytokines after stroke persist significantly longer in aged mice, when compared to young mice. Gut MCs increased at 24 h and 7 days after stroke in aged but not in young mice. Lastly, these changes in HA and MCs in the gut were associated with a shift in bacterial phylum and changes in the beta- (between groups) diversity of the gut microbiota in aged mice when pre-stroke gut luminal samples were compared to post-stroke samples. Together, our results suggest that age-dependent differences in HA signaling and gut MCs contribute to the response to stroke. Pharmacological intervention of MC degranulation or inhibition of the HA-mediated inflammatory cascade might be promising therapy to dampen the prolonged phase of inflammation seen with aging.

## Materials and methods

### Animal experiments

C57BL/6 J young (3 months) and aged (20 months) male mice were housed in a specific pathogen-free facility (light cycle 12/12 h light/dark). Food and water were provided ad libitum. Due to changes in estrous cycle that has impact on histamine levels in young females [32], we used only males for this study.

### Experimental groups

To examine the role of histamine after stroke in young (Yg) and aged (Ag) mice, mice underwent MCAO surgery and were euthanized at 6 h (*n* = 5), 24 h (*n* = 5), and 7 days (*n* = 5) post-ischemia; sham mice received the same surgery, but the suture was not inserted into the MCA (*n* = 3, 5). In total, the groups were divided into Yg-6-sham, Yg-6-stroke, Yg-24-sham, Yg-24-stroke, Ag-6-sham, Ag-6-stroke, Ag-24-sham, and Ag-24-stroke. Seven-day post-ischemic studies were performed only in aged mice (Ag-7d-Sham and Ag-7d-Stroke). A mortality of 20% was observed in the aged 24 h MCAo cohort additionally, 40% mortality was seen at 7 days in aged MCAo mice.

### Middle cerebral artery occlusion

Animals in the respective groups underwent transient focal ischemia under isoflurane anesthesia for 1 h by occlusion of the right middle cerebral artery (MCA). Body temperature was maintained at 37.0 ± 1.0 °C throughout the surgery by an automated temperature control feedback system. One hour after middle cerebral artery occlusion (MCAo), animals were re-anesthetized, and reperfusion was established by the withdrawal of the monofilament. Animals were placed in a recovery cage. All mice were given subcutaneous injections of 0.9% sodium chloride twice a day for 7 days and were provided with wet mash in their cages. Body weight was recorded daily for the duration of the experiments. All experiments were performed by investigators blinded to animal groups and treatments to reduce experimenter bias (Blinded: Due to the size of these animals and stroke having impact on how the animals behave, we used the following methods to blind our samples and the analysis. We followed the blinding of the harvested samples. Tissue harvesting was performed by a blinded individual and analysis is performed by a separate individual who is also blinded. For IHC, the histology core processed the embedding and sectioning of the samples and was performed blinded. Staining was performed and followed up with imaging and analysis blinded. Only after the quantification we un-blinded the samples to allocate them to the respective groups).

### Immunohistochemistry

Formalin fixed, paraffin-embedded intestinal (cecal, ileum) tissue sections (4 μm) were incubated overnight at 4 °C with a primary antibody targeting the mouse antigens (1) histamine receptor 2 (AHR-002, Alomone labs, Israel), (2) Tryptase (ab2378, Abcam, USA), after antigen retrieval according to the manufacturer’s instructions. Samples were washed and subsequently incubated with secondary antibody for 45 min at RT (Histofine simple stain anti-rabbit, 414341F, Nacalai, USA and Alexa fluor® 647, ab150131, Abcam, USA). Sections were counter-stained either with the diaminobenzidine substrate kit (Nacalai, USA) followed by hematoxylin, or sections were stained with 6-diamidino-2-phenylindole (DAPI, Thermo Fisher, USA) as previously described for visualization of cell nuclei.

### Lectin staining to visualize intestinal mucus secreting goblet cells

Mouse cecal segments were fixed in Carnoy’s fixative, embedded in paraffin, and serially sectioned to 5-μm sections. Section was stained with hematoxylin and eosin (H&E) for intestine architecture. Terminal mucin glycans were examined using a panel of FITC-conjugated lectins: *Ulex europaeus* agglutinin-1 (UEA-1) for terminal fucose; concanavalin A (CONA) for mannose, *Dolichos biflorus* agglutinin (DBA) for N-acetylgalactosamine, peanut agglutinin (PNA) for galactose, and wheat germ agglutinin (WGA) for N-acetylglucosamine (Vector Laboratories, Burlingame, CA) as previously described [33]. Briefly, de-paraffinized sections were incubated with citrate buffer pH 6 (Vector Labs) for 20 min in a pressure cooker and blocked with PBS containing 10% BSA. Sections were then stained in a humidified chamber with FITC-labeled lectin (10 μg/ml) for 1 h at room temperature. Sections were washed with PBS, counterstained with DAPI (Thermo Fisher, USA) for 5 min at room temperature, and mounted using aqueous mounting media (Sigma Aldrich). Sections were analyzed by confocal microscopy (Leica Dmi8), and fluorescence was semi-qualitatively calculated by tabulating mean pixel intensity using the ImageJ software (National Institutes of Health).

### mRNA in situ hybridization of intestinal tissues

mRNA in situ hybridization (ISH) was performed on the distal ileum part of intestinal tissue from the sham and stroke cohort by using the RNAscope 2.5 HD assay system (Advanced Cell Diagnostics, Hayward, CA) with recommended probes [Probe-Mm-Hrh1 (Catalog # 491141), Probe-Mm-Hrh2 (catalog # 517751), RNAscope 2.5 HD reagent kit-Red]. ISH scores were generated at × 200 magnification and recorded using the RNAscope system counting guidelines: number of purple dots (positive stain for H2R mRNA) per villi. Each point represents data from 15 crypts per section per sample.

### Fluorescence in situ hybridization

For intestinal tissue preparation, the small and large intestines were carefully removed immediately following euthanasia and rapidly dissected. Mouse terminal ileum (3–6 cm above the cecum), cecum, and mouse proximal and distal colon were carefully removed, fixed in 10% formalin fixative at room temperature for 24 h followed by 70% ethanol transfer, then rinsed in 100% ethanol, and embedded in paraffin wax. The tissues were sectioned at a thickness of 4 μm. These sectioned tissues were used for fluorescence in situ hybridization (FISH) staining as described. Four-micrometer sections were mounted on glass slides, baked at 60 °C for 1 h, then de-paraffinized with xylene, and dehydrated in 100% ethanol followed by incubation in ddH2O. A previously validated, 5′ Cy3′-labeled, *EUB*338 bacteria-specific probe (Bact338; 5′- GCTGCCTCCCGTAGGAGT-3′) which is complimentary to the V1 to V4 region of the 16S rRNA gene that is highly conserved in bacteria domain. The probe was hybridized to the samples by adding 20–25 μL of 1:25 dilution probe with hybridization buffer to each slide and placed in a hybridization chamber at 51 °C for overnight. Nuclei were labeled with DAPI. Intestinal sections from mice in each cage (total 5–6 per group) were utilized to confirm bacterial location. The slides were analyzed using a Leica DMi8 confocal microscope (Leica biosystems, USA) equipped with appropriate filter set for Cy3′ fluorescence (ex 550 nm/em 570 nm).

### Intestinal content collection and 16S rRNA gene sequencing

Microbiota in the intestinal (cecum and feces) samples were collected from mice and stored in sterile tubes at − 80 °C until analyzed. Bacteria taxa in each intestinal content samples were analyzed by amplifying the V4-V5 hyper-variable regions of the 16S rRNA gene using high throughput sequence analysis (Illumina MiSeq platform). Quality filtered 16S rRNA sequences were clustered into operational taxonomic units (OTUs), with 97% similarity, by closed reference OTU-picking using the UCLUST algorithm and GreenGenes reference database (v13.5) as implemented in Quantitative Insights Into Microbial Ecology (QIIME versions 1.6 and 1.7). Sequences were checked for chimeras using ChimeraSlayer with standard options as implemented in QIIME. Sequences not clustered were identified using the Ribosomal Database Project to the lowest possible taxonomic level. The data were randomly rarefied to 10,000 sequences per sample prior to any downstream analysis.

### Murine cytokine measurements in blood plasma by protein multiplex

Relative amounts of GM-CSF, IFN-gamma, IL-1, IL-6, IL-9, IP-10/CXCL10, MCP-1/CCL2, VEGF-A, and G-CSF in the blood plasma were measured using cytokine multiplex kits (Millipore, Billerica, MA, USA). Quantification of cytokines was performed using the MAGPIX system (Austin, TX, USA) according to the manufacturer’s instructions. Briefly, 25 μl of plasma samples collected from each mouse were thawed completely and diluted with the same amount of Assay Buffer provided in the kits. The assays were performed in duplicate blindly. The reports generated by the MILLIPLEX® Analyst 5.1 Software were carefully reviewed and only cytokines that were within the limit of detection value and below the saturated value were considered. The detection limits for the aforementioned cytokines were between 10,000 pg/ml and 3.20 pg/ml, respectively.

### mRNA gene expression in the intestinal mucosa

To quantify relative mRNA expression levels of histamine receptor 1 and 2 (H1R and H2R), interferon (IFN)-γ, tumor necrosis factor (TNF)-α, interferon gamma-inducible protein (IP)-10, interleukin (IL)-6, IL-1, and IL-12, RNA was extracted from intestinal mucosa samples (cecum) using the miRNeasy® mini kit (QIAGEN). One microgram of RNA was reverse-transcribed to single-stranded cDNA using the RevertAid H minus First Strand cDNA Synthesis Kit (Thermo Fisher, USA). Reverse transcriptase real-time (RT) PCR was performed using the Quant Studio 3 Real-Time PCR system (Applied Biosystems, USA). The RT-PCR reaction mix (adjusted with H_2_O to a total volume of 20 μl) contained 1 μl template DNA, 10 μl Power SYBR Green PCR master mix (ABI), and 0.5 μl of the respective primers (10 μM each). The forward and reverse primers used for IFN-γ, IP-10, IL-12, IL-17, TNF-α, and IL-6 quantification were described previously [5, 6]. Relative mRNA target gene expression levels (Ratio = [(E_target_) ^dCPtarget (control-sample)^]**/**[(E_ref._) ^dCPref. (control-sample)^]) were normalized to the house keeping gene glyceraldehyde 3-phosphate dehydrogenase (GAPDH) and used as a reference. Subsequently, intestinal mucosal cytokine of the sham control group was set to 1.0 and used as the calibrator to identify the relative mRNA fold difference between the sham and stroke groups at 6 h, 24 h, and 7 days after stroke.

### Toluidine blue staining in human autopsy brain

Formalin-fixed paraffin-embedded human brain autopsy sections were cut at 30 μm. The slides were deparaffinized and hydrated. Slides were treated with toluidine blue working solution (1% toluidine blue ethanol solution in 1% sodium chloride) followed by dehydration. Nuclei were counterstained with hematoxylin. Human brain autopsy samples were obtained from stroke patients. The infarct region is from the cortex. MCs were quantified by counting the positively stained cells around the infarct region per section with a × 40 magnification.

### Flow cytometry

#### Brain

After removal of intestinal tissue, mice were transcardially perfused with 60 ml cold, sterile PBS prior to aseptic removal of the spleen, lung, and brain tissues. Brain tissue was placed in complete Roswell Park Memorial Institute medium 1640 (Lonza) medium and mechanically and enzymatically digested in Collagenase/Dispase (1 mg/mL) and DNase (10 mg/mL; Roche Diagnostics) for 45 min at 37 °C. Lung tissue was processed similarly with the exception that digestion cocktail contained hyaluronidase (MilliporeSigma, 3000 U/digestion), as well. The cell suspension was filtered through a 70-μm filter. Leukocytes were harvested from the interphase of a 70-to-30% and 70-to-40% Percoll gradients for the brain and the lung tissues, respectively. MCs were gated for CD45 positive (+) followed by FCεR1^+^ with CD117 (c-Kit^+^) expression.

#### Intestines

Tissue-specific protocols were used to obtain single-cell suspensions. Following the euthanasia by Avertin injection, the small (ileum) and large (cecum and proximal colon) intestines were rapidly removed and placed in ice-cold PBS. The intestinal tissue was opened longitudinally after removal of fat and connective tissues. Fecal content was removed and the tissue was cut into pieces (approximately 1.0 cm) after washed in ice-cold PBS. Intestinal tissues were then incubated in 5 mL of 5 mM ethylenediaminetetraacetic acid (EDTA) in Hank’s Buffered Salt Solution (HBSS, Invitrogen, Carlsbad, CA) for 30 min at 37 °C with slow rotation (100 rpm). The epithelial cell layer was removed and filtered through 70-μm cell strainers. The retrieved intestinal pieces were washed in HBSS and cut into smaller pieces and immersed in 10 mL digestion solution containing 5% FBS (Sigma-Aldrich, St. Louis, MO), collagenase IV (1.75 mg/mL; Roche, Nutley), and DNase I (0.5 mg/mL; Sigma-Aldrich) at 37 °C for 45 min with slow rotation. MCs were gated for CD45 positive (+) followed by FCεR1^+^ with CD117 (c-Kit^+^) expression.

### Mass spectrometry histamine quantification

Histamine concentrations were quantified in the blood plasma at 6-h, 24-h post-stroke, and sham control of Yg and Ag mice. Blood plasma was processed through methanol (Sigma Aldrich, USA) separation. The obtained supernatants were transferred into 3-kDa filtrate and centrifuged for 14,000 *g*, 40 min at room temperature. Flow-through was collected, and mass spectrometry analysis was performed. Mass spectrometric quantification was performed as follows.

Histamine, formic acid (FA), and perfluoroheptanoic acid (PFHA) were obtained from Sigma-Aldrich (St. Louis, MO). Histamine-α,α,β,β-d4 was obtained from CDN isotopes (Pointe-Claire, Canada). Water and acetonitrile (ACN) were obtained from Fisher Scientific (Waltham, MA). The histamine-d4 internal standard solution was prepared at a concentration of 250 ng/mL of d4-histamine in water with 0.1% FA. Thirty microliters of internal standard solution was added to 30 μL of each sample, vortexed for 1 min, and dried in a SpeedVac for 5 h. Thirty microliters of water:0.1% FA was added to each sample, vortexed for 1 min and centrifuged for 5 min at 10,000 rpm. Samples were then loaded into 0.5 mL autosampler vials for quantification.

Chromatography was performed on a Shimadzu (Kyoto, Japan) Nexera-XR HPLC system consisting of a SIL-20ACxr autosampler, a CTO-20 AC column oven, and 2 LC-20ADxr binary pumps. Samples were loaded onto a Phenomenex (Torrance, CA) 1 mm × 50 mm phenylhexyl reversed-phased column equipped with a Phenomenex phenylhexyl 4 mm × 2 mm guard column. The aqueous mobile phase (A) consisted of H_2_O:ACN:FA:PFHA (99.3:0.5:0.1:0.1 v/v/v/v), and the organic mobile phase (B) consisted of H_2_O:FA (99.9:0.1 v/v). Column flow was 80 μL/min, and 5 μL of sample was injected onto the column and eluted with a constant mobile phase flow rate of 80 μL/min. The elution gradient was optimized as follows: started from 10% B and increased to 70% B over 5 min; ramp to 80% B for 6 s and held for 1 min; ramp back to 10% B over 6 s and maintained at 10% for a total chromatographic run time of 12 min to re-equilibrate.

Selected reaction monitoring (SRM) was performed on a Sciex (Framingham, MA) 6500 QTRAP with a Turbo V source. The mass spectrometer was operated in the positive ion mode under the following conditions: curtain gas 20 psi; collision gas: HIGH; spray voltage 4.5 kV; ion source gas 1 20 psi; ion source gas 2 20 psi; interface heater temperature 175 °C; Q1 and Q3 resolution: unit; scan time 100 mS; declustering potential 100 V; entrance potential 8 V; collision exit potential 10 V. The instrument was calibrated by using Sciex PPG calibration standard and tuned to the manufacturer’s specifications. SRM transitions monitored for histamine were 112 → 95 (20 eV) and 112 → 68 (30 eV). For histamine-d4, the SRM transitions 116 → 99 (20 eV) and 116 → 72 (30 eV) were monitored. Data were acquired with the Analyst® Software (ver 1.6.2) and quantification performed using the Multiquant™ Software (ver 3.0.1).

### Statistics

Data were tested for normal distribution using the Kolmogorov–Smirnov test. Normally distributed data are presented as means with standard deviation while the medians with their range are given for non-normally distributed data. Significance of differences between sham (control) and stroke (experimental) at 6-h, 24-h, and 7-days post-stroke mice were analyzed using the one-way analysis of variance test for normally distributed data (or) the Kruskal-Wallis test for non-normally distributed data, followed by either Bonferroni/Tukey’s multiple comparison post hoc tests. Differences between sham and stroke at single time-point were analyzed using Student’s *t* test followed by the Mann-Whitney test for non-normally distributed data. Differences between the groups were considered significant at *P < 0·05, **P < 0.01, ***P < 0.001. The Prism 5.0 software (Graph Pad Software, Inc., La Jolla, CA, USA) for Windows was used for data presentation and for data analysis. All experiments were performed by an investigator blinded to stroke and age groups during analysis. Differences in Phyla in the gut microbiota of young and aged mice were analyzed using the unweighted UniFrac distance and plotted in a Principal Coordinates Analysis. The UniFrac distance is a measure that takes into account the branch length shared by the young and aged microbiota when placed on a common phylogenetic tree [34, 35].

## Results

### Aging is associated with increased number of mast cells (MCs) in the gut and in the brain

**Fig. 1:**
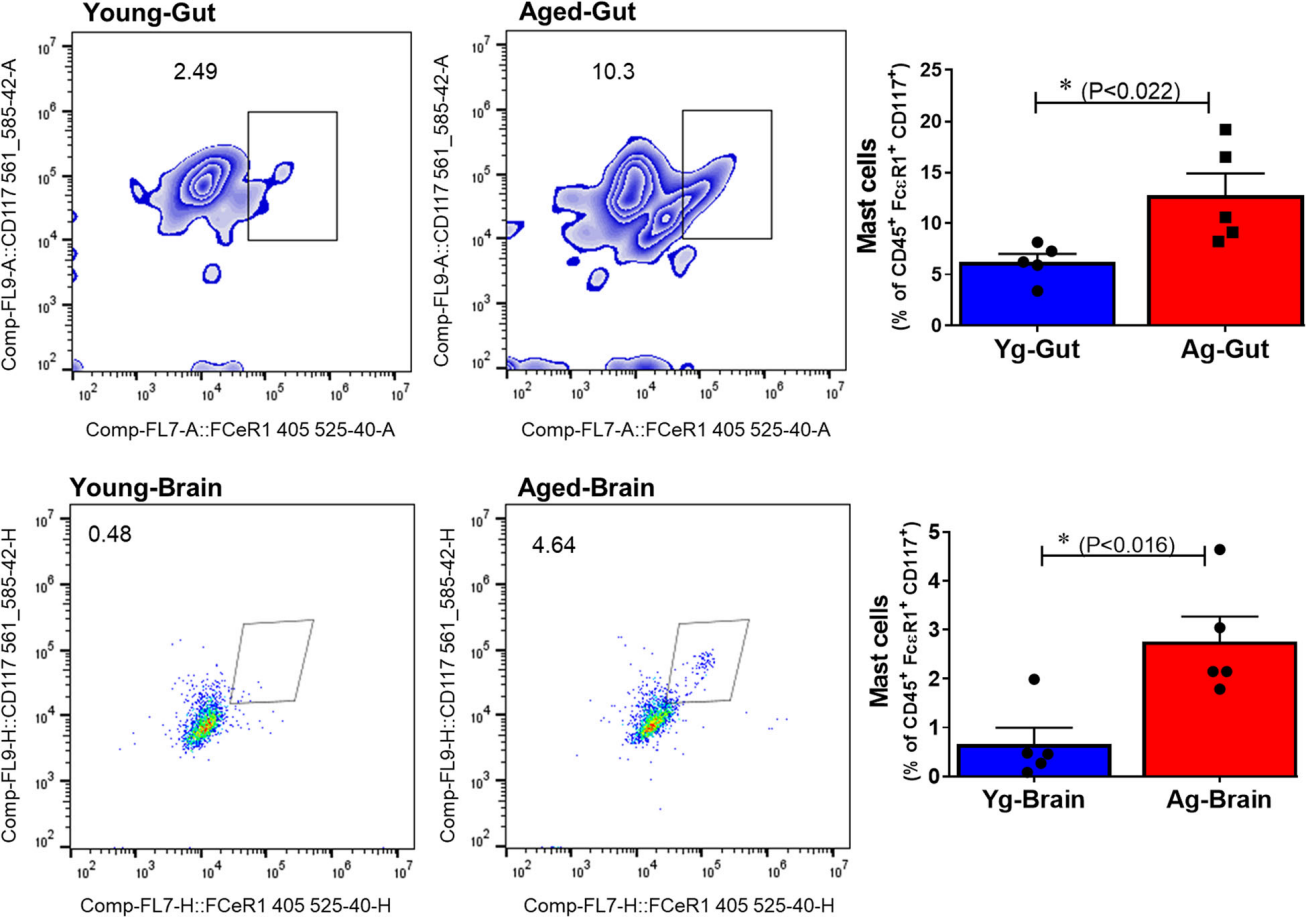
Aging caused two-fold increase in resident MC numbers in both gut and brain of aged compared to young mice. *n* = 5 per group. Data are expressed as mean ± SEM, as well as individual values, and are obtained from two independent experiments at different time points. *P < 0.05, **P < 0.01. *P* values were calculated using two-tailed unpaired Student’s *t* test

Aging is an important risk factor for stroke and is accompanied by low-level inflammation [3, 36]. Aging alters the immunological response to stroke [36, 37]. We investigated if aging has an impact on tissue-specific MC populations and if age-associated differences in MCs play a role in post-stroke inflammation. MC progenitors are housed in the intestinal mucosa. Thus, we first investigated changes in the MC populations in the gut and the brain of naïve Yg (3 months) and Ag (20 months) male WT mice. After exclusion of B cells, T cells, macrophages, and dendritic cells, MCs were identified as CD45^+^ FCεR1^+^ CD117/c-Kit^+^ population. We found a significant increase in relative frequency of MCs, as a percentage of CD45^+^ population, in both the gut and the brain of Ag mice compared to Yg mice (Fig. 1). We then, using human brain samples, investigated the presence of MCs in the infarct area. Human brain autopsy samples from stroke patients showed highly granulated MCs around the infarct area whereas MCs were not detected in young or aged control samples (Supplemental Figure 1). We then determined if the increased number of gut MCs was associated with higher levels of circulating HA after stroke in Ag and Yg mice.

### Stroke leads to elevated HA in the systemic circulation in an age-dependent manner

**Fig. 2:**
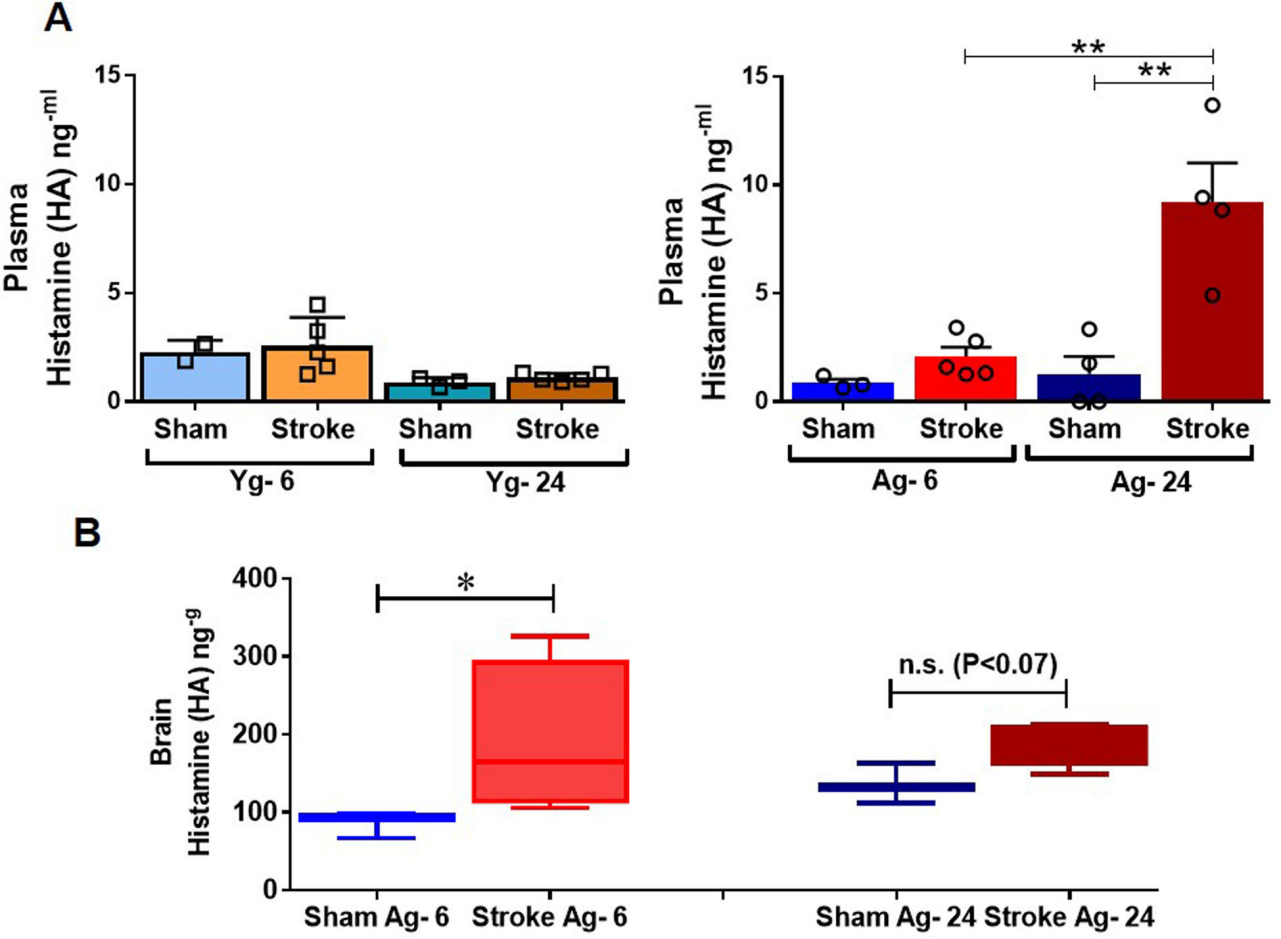
Elevated levels of histamine following ischemic stroke in **a** blood plasma of young (Yg) and aged (Ag) mice at 6 and 24-h post-stroke compared to age matched controls. **b** Histamine levels of brain quantified by mass spectrometry in aged (Ag) mice at 6 and 24-h post-stroke compared to age-matched sham control mice. *n* = 3 (sham) and 5 (stroke) per group. Data are expressed as mean ± SEM (**a**) and median ± range (**b**), as well as individual values. *P < 0.05, **P < 0.01, ***P < 0.001. *P* values were calculated using one-way analysis of variance with Tukey multiple comparisons correction (**a**) and Kruskal-Wallis test with Dunn’s multiple comparison (**b**)

HA is released by resident MCs, as an immediate response to tissue injury and initiator of inflammation [9, 11–16]. We hypothesized that aging leads to increases in systemic HA levels after stroke. We quantified plasma HA levels at 6 and 24 h after stroke in Yg (3 months) and Ag (20 months) mice. Mass spectrometry analysis demonstrated that Yg mice showed no changes in plasma HA levels at 6 and 24 h (Yg: 6-h = 1.12 ± 0.2 and 24-h = 1.10 ± 0.18 ng ml^−1^) after stroke compared to age-matched sham controls. In contrast, Ag mice exhibited an increase in plasma HA levels at 6 h that was significantly increased at 24 h after stroke when compared to age-matched shams (Fig. 2a). In line with these systemic changes, brain HA levels were significantly higher at 6 h after stroke in Ag group when compared to age-matched shams (Fig. 2b). This increased brain HA level was not significant at 24 h, although there was a trend towards increased in HA levels after stroke in the Ag group (Fig. 2b). No changes in brain HA level was observed in the Yg group at either timepoint (data not shown). These findings suggest that aging is associated with increased plasma and brain HA levels after stroke in the acute phase. We speculated that increased systemic HA levels after stroke might also lead to increased HA receptor expression in the gut.

### Stroke leads to elevated gut mucosal H2R expression

**Fig. 3:**
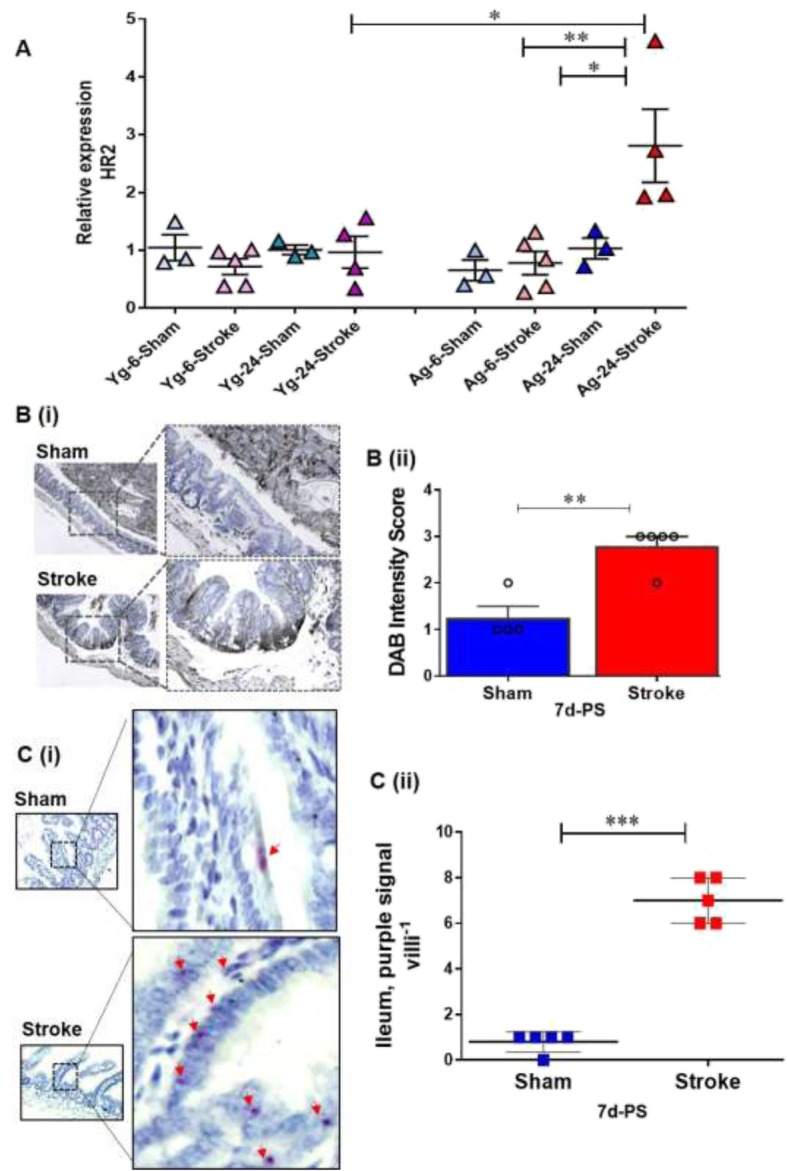
Increased levels of histamine receptor 2 (HR2) measured in the intestinal samples of young and aged mice for 6-h, 24-h, and 7-days post-stroke compared to the age matched controls. **a** mRNA expression levels of HR2 quantified by qPCR in young and aged mice at 6 and 24 h post-stroke. **b** (i) Protein levels of HR2 quantified by IHC followed by image J quantification in aged mice at 7 days post-stroke and **c** mRNA levels of HR2 quantified by RNA in situ hybridization and analyzed by image-J in the intestine of Ag mice at 7 days post-stroke. *n* = 3 (sham) and 5 (stroke) per group (**a**) and 5 (sham) and 5 (stroke) per group (**b** and **c**). Data are expressed as mean ± SEM (**a**), (**b**), and (**c**), as well as individual values. *P < 0.05, **P < 0.01, ***P < 0.001. *P* values were calculated using one-way analysis of variance with Tukey multiple comparisons correction (**a**) or with two-tailed unpaired Student’s *t* test (**b** and **c**). **b** Brown-HR2 protein; blue-Nuclei (**b**) purple dots (red arrows)-mRNA HR2

We then determined whether this increase in systemic HA levels after stroke in Ag but not Yg mice is associated with increased expression of the gut-specific HA receptor, H2R in Ag but not in Yg mice after stroke. We performed mRNA gene expression analysis from gut tissues of Yg and Ag mice at 6- and 24-h post-stroke timepoints. We found that H2R gene expression was significantly increased in the gut mucosa at 24 h after stroke in Ag mice but not in Yg mice, when compared to age-matched shams (Fig. 3a). Since we observed a significant increase in H2R expression at 24-h post-stroke only in aged mice, we performed additional experiments in Ag mice to examine the sub-acute effects of stroke on H2R expression in the gut mucosa. Gut tissue was collected from Ag (20 months) sham and stroke groups at 7-days post-stroke (7d PS). Ag mice showed significantly increased H2R expression in the gut at both the protein and mRNA levels within the intestinal mucosa (Fig. 3b, c). This increased expression was significant in the lamina mucosa where most of the immune cells reside in the gut tissue. RNA in situ hybridization (Fig. 3c) showed increased H2R mRNA expression in the lamina mucosa, which further supports the notion that there is an increase in H2R protein levels after stroke in the gut epithelium of Ag mice at 7 days PS.

### Stroke-induced HA release is associated with increased pro-inflammatory cytokines in both systemic circulation and the gut

**Fig. 4:**
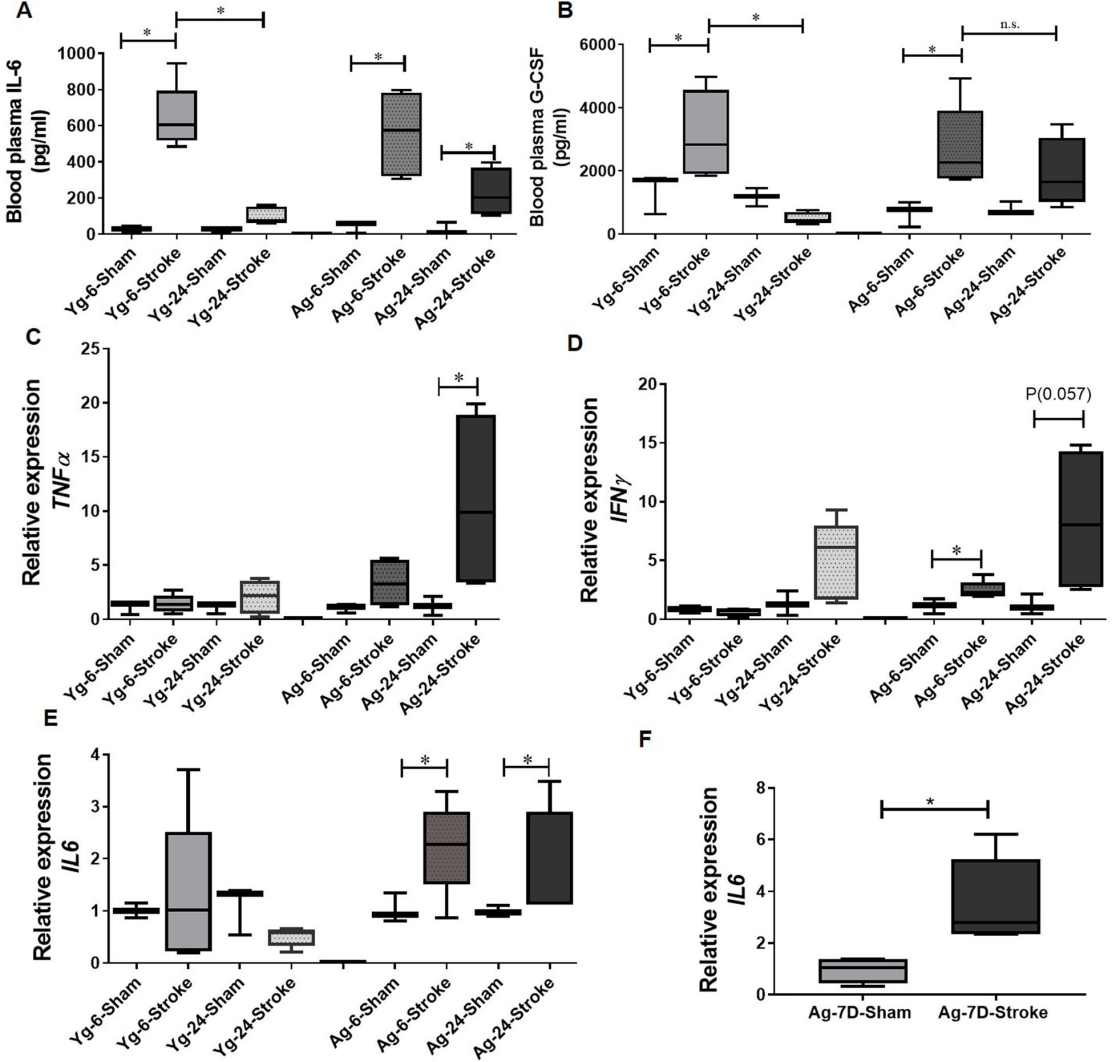
Elevated levels of inflammatory cytokines in the peripheral circulation and gut tissue of young and aged mice at 6 h, 24 h, and 7 days post-stroke compared to the age-matched sham controls. Plasma levels of **a** IL-6 and **b** G-CSF quantified by multiplex at 6 h and 24 h post-stroke in young (Yg) and aged (Ag) mice compared to sham controls. mRNA expression levels of pro-inflammatory cytokines **c** TNF-α, **d** IFN-γ, **e** IL-6 of gut tissue from Yg and Ag mice at 6 and 24 h post-stroke compared to sham controls. **f** mRNA levels of IL-6 in gut tissue of aged mice at 7 days after stroke compared to aged-matched sham controls. *n* = 3 (sham) and 5 (stroke) per group (**a**–**e**) and 5 (sham) and 5 (stroke) per group (**f**). Data are expressed as median with range, as well as individual values. *P < 0.05, **P < 0.01, and ***P < 0.001. *P* values were calculated using one-way analysis of variance with Tukey multiple comparisons correction (**a**–**e**) and two-tailed unpaired Student’s *t* test, Mann-Whitney correction (**f**).

IL-6 is a key pro-inflammatory molecule and mediates stroke-induced inflammation [38–40]. IL-6 increases MCs maturation [41] by inducing the FcεR1 receptor on MCs [42–45] and by upregulating HA production. In young mice, we found increased levels of plasma IL-6 at 6-h post-stroke that normalized by 24 h, when compared to age-matched shams. In aged mice however, a significant increase in plasma IL-6 levels observed at 6 h persisted up to 24 h after stroke, when compared to age-matched shams (Fig. 4a).

Our data showed that granulocyte colony-stimulating factor (G-CSF) was significantly increased in the plasma at 6 h after stroke in both Yg and Ag mice compared to age-matched shams. However, there was a significant reduction in plasma G-CSF by 24 h after stroke only in Yg but not in Ag mice, when compared to age-matched shams (Fig. 4b).

In the gut, IL-6 and TNF-α gene expression levels were significantly higher at 6-h and 24-h post-stroke in Ag mice compared to age-matched controls (Fig. 4c, e). This increase was not seen in Yg mice (Fig. 4c, e). IFN-γ expression was significantly increased at 24 h after stroke in Ag mice whereas Yg mice had no change in IFN-γ levels at either timepoints when each group was compared to their age-matched shams (Fig. 4d). Interestingly, IL-6 mRNA levels were significantly elevated in the gut even at 7 days after stroke in aged mice compared to the age-matched sham controls (Fig. 4f). These findings support our hypothesis that increased HA and pro-inflammatory cytokines increases in both the plasma and in the gut of aged animals at 24 h after stroke, when compared to young cohorts.

### Stroke leads to increased number of gut MCs

**Fig. 5:**
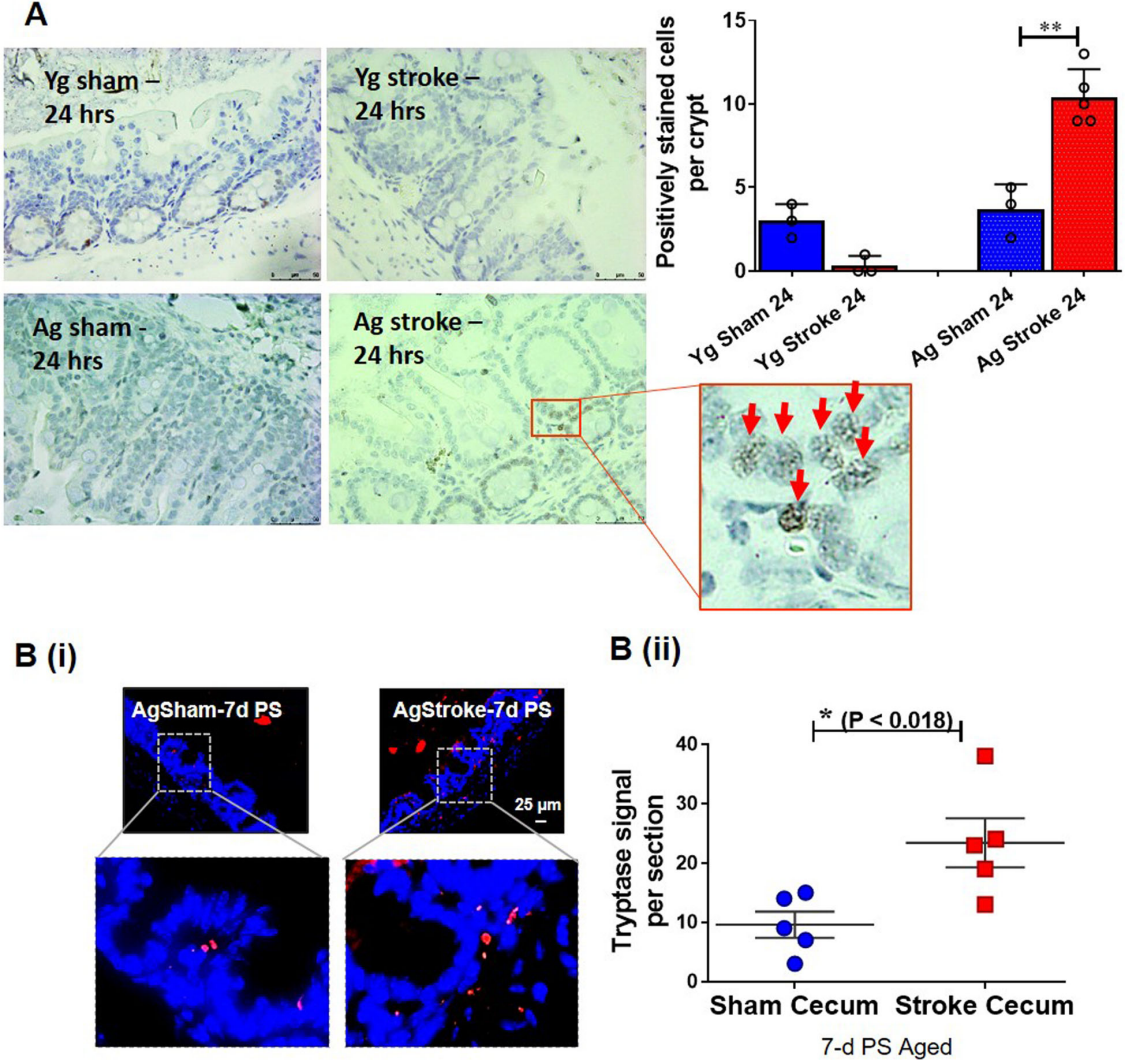
Increased mast cell (MC) numbers in the gut mucosa measured in the intestinal samples of young and aged mice for 6 h, 24 h, and 7 days post-stroke compared to the age matched controls. **a** MCs quantified by immunohistochemistry in the intestinal samples of young and aged mice at 6-h and 24-h post-stroke compared to their age-matched sham controls. **b** MCs quantified by fluorescence immunohistochemistry in the intestinal samples of aged mice at 7-days post-stroke and **c** mRNA levels of HR2 quantified by RNA in situ hybridization and analyzed by image-J in the intestine of Ag mice at 7-days post-stroke. *n* = 3 (sham) and 5 (stroke) per group (**a**) and 5 (sham) and 5 (stroke) per group (**b**). Data are expressed as mean ± SEM (**a**) and (**b**), as well as individual values. *P < 0.05, **P < 0.01, ***P < 0.001. *P* values were calculated using one-way analysis of variance with Tukey multiple comparisons correction (**a**) and two-tailed unpaired Student’s *t* test (**b**). **a** Red arrows-Mast cell; **b** Red-mast cells

MCs are a primary source of HA during acute inflammation. Mucosal MC progenitors constitutively home to the intestinal mucosa and are recruited, then mature, after inflammatory stimuli [19, 20]. To investigate the cause of increased H2R expression, we examined the number of gut MCs after stroke in aged mice. Using immunohistochemistry, we found that young mice show no detectible levels of gut MCs 24 h after stroke (Fig. 5a). However, a significant increase in gut MCs was seen at 24 h after stroke in aged mice compared to age-matched shams (Fig. 5a). An increase in the gut MCs, assessed by tryptase signal intensity, was also present 7 days after stroke in aged mice, when compared to age-matched shams (Fig. 5b).

### Stroke is associated with reduction in mucus-secreting goblet cells in the gut

**Fig. 6:**
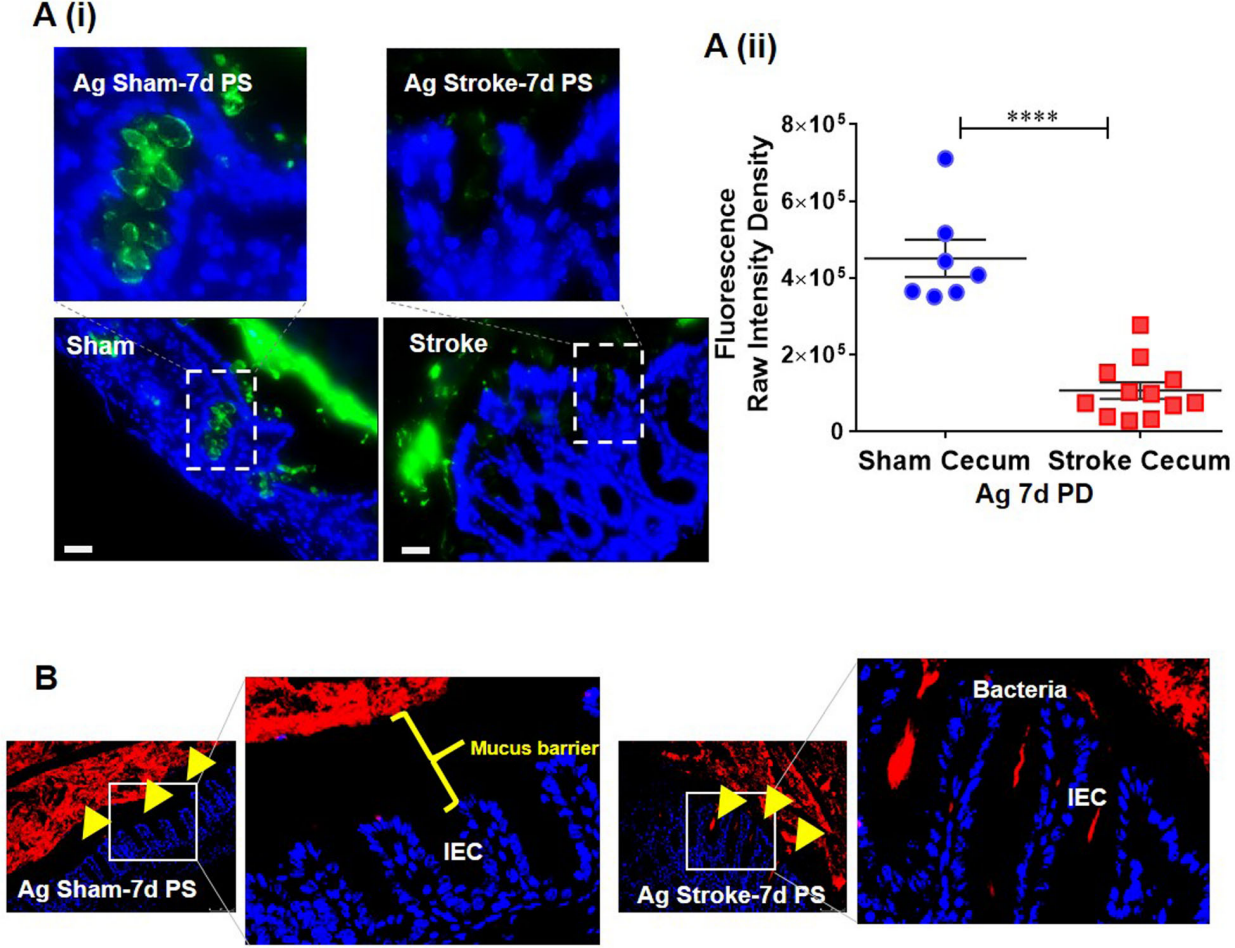
Reduced fucosylated goblet cells and mucus barrier of the gut mucosa measured in the intestinal samples of aged mice 7-days post-stroke compared to the age-matched controls. **a** Goblet cells analyzed by immunohistochemistry and quantified by image-J in the intestinal samples of aged mice at 7-days post-stroke compared to their age-matched sham controls. **b** FISH staining showing bacterial growth and mucus thickness of gut tissue measured by confocal microscopy in the intestinal samples of aged mice at 7-days post-stroke. Number of mice used 5 (sham) and 5 (stroke) per group (**a** and **b**). Data are expressed as mean ± SEM (**a**) as well as individual values. *P < 0.05, **P < 0.01, ***P < 0.001. *P* values were calculated using two-tailed unpaired Student’s *t* test. **a** Green-fucosylated goblet cell and mucus; **b** red-Bacterial biofilm (**a** and **b**) Blue-DAPI nuclei stain

Mucus is secreted by the goblet cells of the gut epithelium and is highly glycosylated [46]. In the presence of inflammation, mucus fucosylation is significantly depleted [8, 47]. To follow up on our results showing increased gut inflammation in aged mice, we assessed the mucus barrier integrity by quantifying the amount of fucosylated mucin in aged mice. Our results from aged mice at 7-days post-stroke showed a significant reduction in goblet cells filled with fucosylated mucus, when compared to shams (Fig. 6a). Reduced mucus barrier integrity was also associated with increased bacterial breach observed by fluorescence in situ hybridization of the gut mucosa (Fig. 6b). These findings suggest that increased gut inflammation in aged mice is associated with a loss of gut barrier integrity, which might explain the persistence of post-stroke inflammation in aged animals.

### Increased H2R and MCs after stroke are associated with dysbiosis of the gut microbiota

**Fig. 7:**
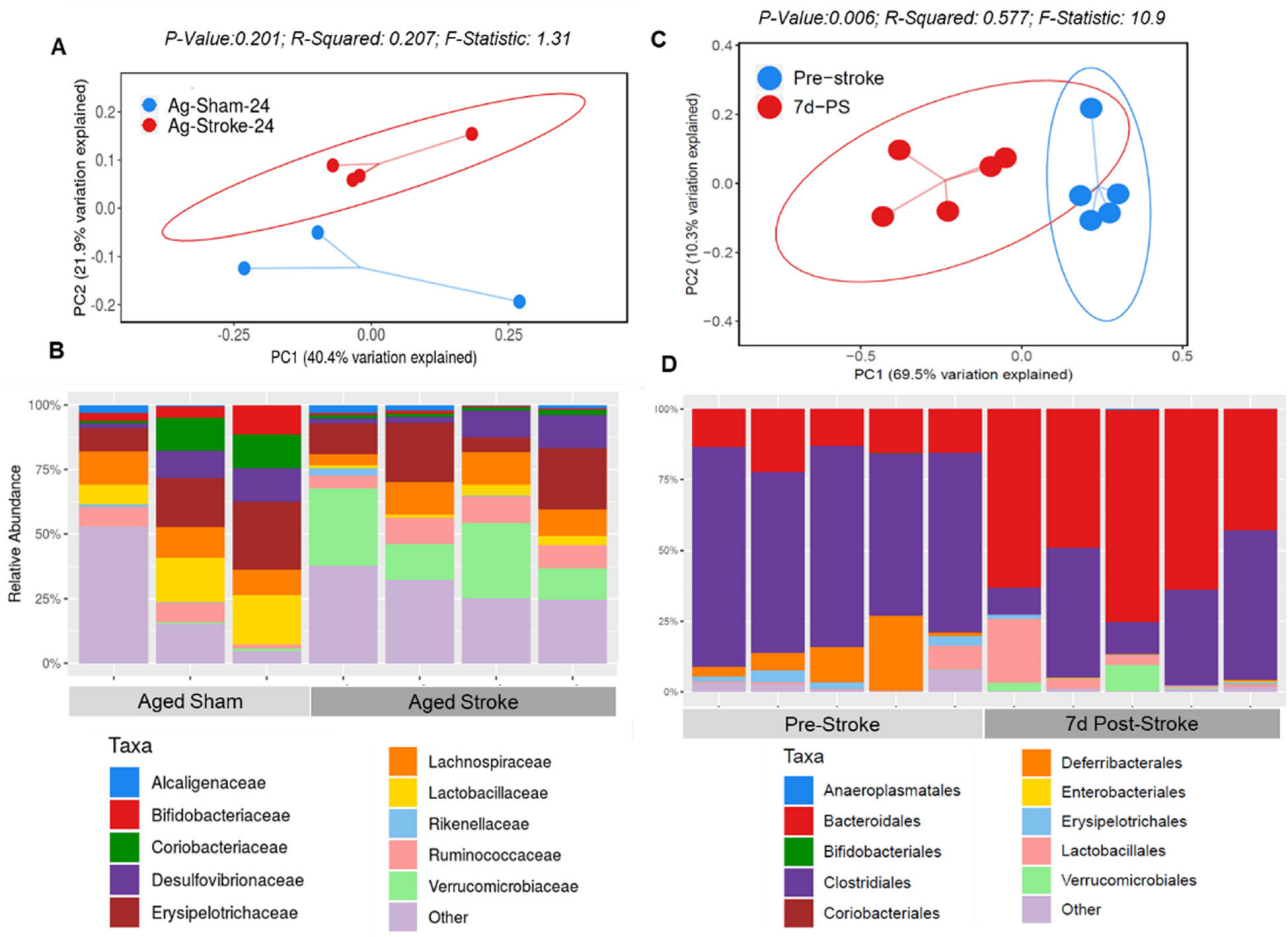
Compositional differences in gut microbiota by 16S rRNA sequencing and qPCR of intestinal luminal content. **a** Visualization of beta-diversity, or between-samples diversity, with weighted UniFrac distances by principal coordinate analysis (PCoA) shows a clustering effect by strain between aged mice at 24-h post-stroke compared to age-matched sham controls and **b** corresponding family level bacterial distribution. **c** Visualization of beta-diversity, or between-samples diversity, with weighted UniFrac distances by principal coordinate analysis (PCoA) shows a clustering effect by strain between aged mice at 7-days post-stroke compared to age-matched sham controls and **d** corresponding order level bacterial distribution. *n* = 3 sham and *n* = 5 stroke (**a** and **b**); *n* = 5 sham and *n* = 5 stroke (**c** and **d**)

The composition of gut microbiota can be influenced by intestinal inflammation [48]. We performed 16S rRNA sequencing on gut contents to examine alternations in the microbial diversity caused by stroke-induced inflammation in the Yg and Ag mice at 6 h, 24 h, and 7 days after stroke and compared these to age-matched sham controls. Consistent with the increased H2R expression levels and MCs in the gut, aged mice showed a shift in the beta-diversity or between-samples diversity, with weighted UniFrac distances by principal coordinate analysis (PCoA), as early as 24 h after stroke compared to age-matched controls (Fig. 7a). Interestingly, we observed an increase in Verrucomicrobiaceae family in aged mice at 24 h after stroke compared to age-matched shams (Fig. 7b). These changes were not seen at the earlier timepoint of 6 h in aged or young (6 and 24 h) stroke mice. The alpha-diversity, or within-sample diversity, was not different between sham and stroke mice 7-days post-stroke (not shown). Upon visualization of beta-diversity, or between-samples diversity, with weighted UniFrac distances by PCoA, a significant clustering effect (*P* = 0.006) emerged along the PC1 axis (69.5% variation explained) in 7-days post-stroke Ag mice luminal content compared to pre-stroke Ag mice (Fig. 7c). Closer examination of 16S data at the order level showed a significant reduction of Clostridiales and an increase in Bacteroidales in the aged mice 7-days post-stroke compared to pre-stroke mice (Fig. 7d). Overall, our 16S data show that compositional differences in the gut microbiota exist as early as 24 h after stroke and continued at 7 days after stroke in aged mice, when compared to non-stroke samples (Fig. 7c, d).

## Discussion

Histamine (HA) is an important signaling molecule secreted from resident MCs [8, 9] and is necessary for MC maturation [14]. MC progenitors are housed in the gut mucosa and migrate to the site of inflammation upon activation [19, 20]. MCs are early responders and are involved in the acute blood-brain barrier changes after cerebral ischemia and hemorrhage [49]. Therefore, we hypothesized that stroke would increase gut MC activation, leading to an elevation in systemic HA levels and an increase in peripheral inflammation. Elevated MC signaling is known to increase inflammation due to increased HA release and HR expression [8, 9]. Our results demonstrated that stroke leads to an increase in the gut MC population in aged mice as early as 6-h post-stroke. HA is an important signaling molecule from resident MCs [8, 9]. Our data support the assumption that an increase in gut MCs leads to an increase in systemic HA and H2R expression levels after stroke in Ag mice. In line with the elevation in H2R expression, we found elevated IL-6 expression levels in the gut mucosa after stroke only in Ag mice. IL-6 is a pro-inflammatory molecule and mediates stroke-induced inflammation [38–40] and also increases MC maturation [41]. IL-6 also upregulates histamine production and induces the FcεR1 immunoglobulin receptor found on mast cells [42–45]. In addition, we found that mucus secreting fucosylated goblet cells were reduced after stroke in Ag mice. Fucosylation of mucus is a major determinant of a healthy gut, and reduction in fucosylation is associated with gut inflammation [5, 8, 50, 51]. Of note, these changes were only examined in male mice, as estradiol induces partial release of MCs [52]. As the estrous cycle varies every 4 days in young female mice [32], we focused these initial studies on male mice. Evaluation of aged females would add translational value to these findings in future experiments.

Histamine release is an immediate response from MCs after injury, and this triggers the production of a variety of pro-inflammatory molecules [9–16]. Previous work has shown that an increase in systemic HA levels occurs with aging [30]. In association with altered HA levels, changes in IL-1β and TNFα in the brain have also been reported [53]. Similarly, we observed elevated HA levels in the brain of Ag mice acutely after stroke (6 h) but not at the sub-acute timepoint of 24 h. In contrast, post-stroke HA levels remain high at both acute and sub-acute phases in the plasma of Ag mice. We then investigated the role of the gut in the stroke-induced elevation of HA. Previous studies have shown that gut mucosal MCs (gMCs) are a major source of HA [17, 18]. Interestingly, mucosal MC progenitors constitutively home to the intestinal mucosa and are recruited and matured during inflammation [19, 20]. Importantly, our data showed a basal increase in resident MCs in the brain and gut of aged mice compared to young mice. Therefore, we believe that the increase in systemic HA induced by stroke is secondary to the increased gut MC population in Ag mice. In support of this hypothesis, we observed a significant increase in the gut MCs at 24 h after stroke and found this effect to be age-dependent.

Reducing HA signaling may have therapeutic potential. Administration of a H2R antagonist was associated with preserved stroke volume and reduced risk for incident heart failure [54]. Others have found that administration of ranitidine, a H2-receptor antagonist, reduced neuronal death induced by oxygen-glucose deprivation in an in vitro model of ischemia [55]. In line with these observations, we found a significant increase in H2R expression in the gut as early as 24 h and as late as 7 days after stroke in aged mice. Therefore, we speculate that stroke increases gut MCs, which contributes to severe peripheral inflammation via increased gut HA-H2R activation in Ag mice. In line with these findings, we found that pro-inflammatory cytokines, such as IL-6, TNF-α, and IFN-γ were increased in the gut mucosa of aged mice after stroke. Similarly, we found increased systemic pro-inflammatory cytokines IL-6 and G-CSF levels in aged mice at both 6 h and at 24 h after stroke.

A previous study has reported that MCs derived from meninges worsen damage in murine models of stroke [29]. However, this study used global MC knockout mice that had MCs deleted from all tissues. Therefore, the role of peripheral MCs, specifically those from the gut where most progenitor MCs are found, was not studied. This study is the first evidence that shows the importance of peripheral MCs and their role in stroke in aged animals. Our group has previously shown that stroke leads to increased neuroinflammation in aged animals [35, 36, 56]. We have also shown that the gut is “inflamed” after stroke [35]. However, the initial trigger that connects the brain-gut axis in the response to stroke is not understood. MCs are abundant in the gastrointestinal mucosa [20], and their activation is a primary response to tissue injury [57]. Stroke induces HA accumulation and causes MC degranulation in the neonatal brain [4]. Interestingly, MCs are also early responders in the regulation of acute blood-brain barrier changes after cerebral ischemia and hemorrhage [49] and likely play a key role in the response to injury. In keeping with our hypothesis, we observed an increase in gut MCs, gut H2R expression, and circulating HA levels after stroke with age. These changes were not seen in young mice after stroke, reinforcing the value of examining aged mice in experimental stroke studies. This is important as stroke mainly affects older individuals [58]. We believe that the increase in gut MCs at the sub-acute phase of 24 h might be an important link in the brain-gut axis and the response to stroke. Future studies to validate these findings with knockout mice are needed to confirm the role of HA and gMCs in post-stroke inflammation. In addition, previous research in our lab has shown changes in infarct volume in aged and young male mice 24-h post-stroke. However, the increased infarct volume and neurological scores observed in young post-stroke mice showed reduced inflammation (measured by T cells, monocytes, and microglia activation) compared to aged post-stroke mice that showed reduced infarct volume with increased inflammation in the brain [59]. In addition, our lab has previously demonstrated that aged mice brain develops higher hemorrhage transformation than young mouse brain post-stroke [36]. There is a growing body of evidence that inflammatory cell infiltration is predominantly deleterious in the early phase of ischemia with age [60]. MCs, best known as first responders and pro-inflammatory effector cells, play critical roles in the development of inflammation in many disease settings [11]. Previous studies show that the majority of MCs reside in the gut mucosa [57]. Gut pathology leads to increased activation of MCs [61]. In the current study, we have seen increased gut inflammation with changes in gut microbiome. There is evidence demonstrating that the severity of peripheral inflammation is key to determining post-stroke recovery [62]. In addition, previous studies have shown that ischemia causes reduced levels of diamine oxidase (DAO) that controls histamine availability [63]. However, aging itself reduces basal gut mucosal DAO levels [64] leading to increased HA levels from MCs in Ag but not in Yg*.* Since MCs are immediate responders in the injured site, it is of importance to look at histamine levels and mast cell activation post-stroke. Primarily, aged MCs are known to be in an increased state of activation [31]*.* Changes seen in MCs and inflammation observed in the present study are primarily due to aging effects of stroke. A recent study shows that infarct damage was reduced after stroke in MC-deficient mice, and this was thought to be due to deleterious actions of MCs in the brain [29]. However, this study used whole body knock-out mice and did not analyze the importance of peripheral MCs. Therefore, we assume that focusing on suppressing the mast cell activity in the early phase after stroke might reduce inflammation in aged brain with reduced infiltrating immune cells from the periphery and can be used as therapeutic target for stroke outcome and recovery.

Mucus is secreted by goblet cells of the gut epithelium and it is highly glycosylated [46]. In the presence of inflammation, mucus fucosylation is significantly depleted [8]. Increased inflammation causes reduced mucus synthesis [5, 8], allowing luminal bacteria to come in close contact with the gut epithelium triggering further inflammation [47]. The intestinal sections obtained from aged post-stroke male mice at 7 days showed significantly reduced mucus fucosylation in our study. This was associated with increased inflammatory cytokines upregulated in the gut. It is previously known that under inflammatory conditions, goblet cells go through endoplasmic reticulum stress that then leads to loss of goblet cell and behaves like normal epithelial cell [65, 66]. Also, histamine is known to degranulate goblet cells and cause loss of mucus-filled goblet cells [67, 68]. Since we only found increased systemic and gut mucosal inflammation (histamine and MCs) in aged mice, we assessed the mucus barrier integrity by quantifying the amount of fucosylated mucin only in aged mice at 7-days post-stroke to understand the persisting gut dysfunction long after stroke that might have potential impact on poorer stroke outcomes. One may hypothesize that loss of mucus barrier due to inflammatory cascade caused by stroke-induced histamine increase may feedback to further chronic inflammation by increased antigen translocation as seen in Ag stroke.

Our lab has previously demonstrated that gut dysbiosis (imbalanced microbiota composition leading to reduced barrier integrity [69]) plays an important role in the increased peripheral inflammation seen after stroke in aged animals [35]. Significant changes were seen up to 7 days after stroke in aged mice, but not in young mice, re-emphasizing the value of using aged animal models in stroke studies. Similar to the observation presented by Wong et al. [24], the family Verrucomicrobiaceae was the dominant family in the gut of aged mice after stroke. Most bacterial species within this family belongs to *Akkermansia muciniphila*, a mucin degrader. *A. muciniphila* is known to contribute to gut inflammation. This increase in mucin-degrading bacteria may be a link between the loss of protective mucus barrier that was seen after stroke. This work demonstrates that HA-HR levels are upregulated in the gut shortly after stroke and this is restricted to aged animals. Interestingly, human brain autopsy samples from stroke patients showed MC around the infarct area (Supplemental Figure 1). In addition, stroke and transient ischemic patients display significant changes in gut microbiota composition reported earlier independent of the co-morbidities. This might be due to the sudden severe pain and it is known to cause poor bowel moments [70]. Reduction in bowel moments can indirectly induce dysbiotic microbiome [71]. However, inflammation is known to be the primary cause that leads to long-term health defects, and dysbiosis might contribute to this pathological outcome.

## Conclusion

Post-stroke inflammation is a critical determinant of damage and recovery after stroke [72]. HA secretion after MC degranulation may contribute to inflammation via activation of H2R, blood-brain-barrier disruption, and recruitment of other immune cells to the ischemic brain. Our results show that the increase in gut MCs might be an innate immune response connecting the brain and gut after stroke. This study demonstrates that gut MCs and the H2R are upregulated after stroke in an age-dependent manner and are one of the primary events that occur following stroke. Our data highlight the importance of gut immune cells, specifically MCs in examining the peripheral response mediated by the brain-gut axis dysfunction after stroke.

## Supplementary information

**Additional file 1: Supplemental Figure 1.** Visualization of mast cells (MC) by Toluidine blue staining in the infarct area of human autopsy aged stroke brain samples compared to young and age matched controls. Red arrow indicate mast cells in purple stain. (B) Information about human autopsy samples on age, sex, stroke age and MCs found.

## Data Availability

The datasets supporting the conclusions of this article are included within the article and its additional files are available from the corresponding author on reasonable request.

## Abbreviations

MC: Mast cells
gMC: Gut mast cells
HA: Histamine
HR2: Histamine receptor 2
MCAO: Middle cerebral artery occlusion
IL: Interleukin
TNF-α: Tumor necrosis factor alpha
IFN-γ: Interferon gamma
G-CSF: Granulocyte colony stimulating factor
OTU: Operational taxonomic units
