# Supplementary figures and images for: Age-dependent involvement of gut mast cells and histamine in post-stroke inflammation
Source: J Neuroinflammation. 2020 May 19;17:160. doi: 10.1186/s12974-020-01833-1 (PMC7236952; doi:10.1186/s12974-020-01833-1)

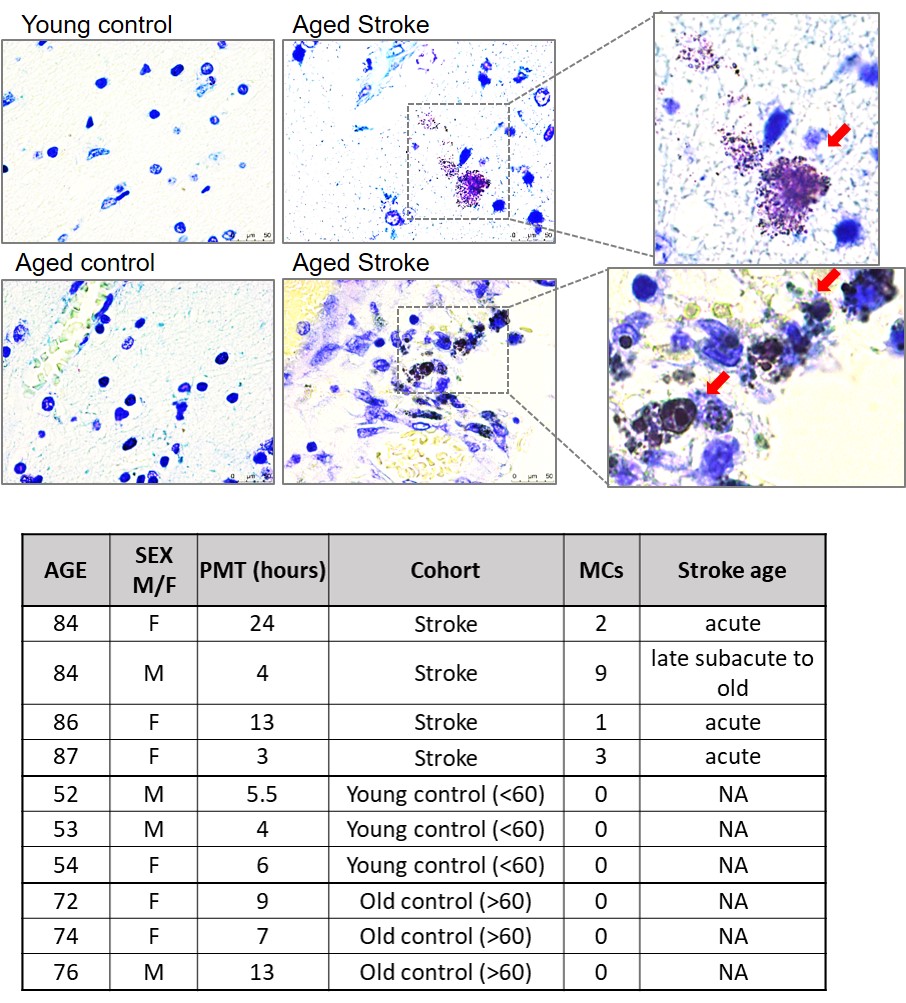

Supplement: Supplementary file 1 — Additional file 1: Supplemental Figure 1. Visualization of mast cells (MC) by Toluidine blue staining in the infarct area of human autopsy aged stroke brain samples compared to young and age matched controls. Red arrow indicate mast cells in purple stain. (B) Information about human autopsy samples on age, sex, stroke age and MCs found. [file 12974_2020_1833_MOESM1_ESM.jpg]
